# Supplementary material for: Cross-Species Transmission of Swine Hepatitis E Virus Genotype 3 to Rabbits
Source: Viruses. 2020 Jan 2;12(1):53. doi: 10.3390/v12010053 (PMC7019366; doi:10.3390/v12010053)
Supplement: Supplementary file 1 [file viruses-12-00053-s001.pdf]

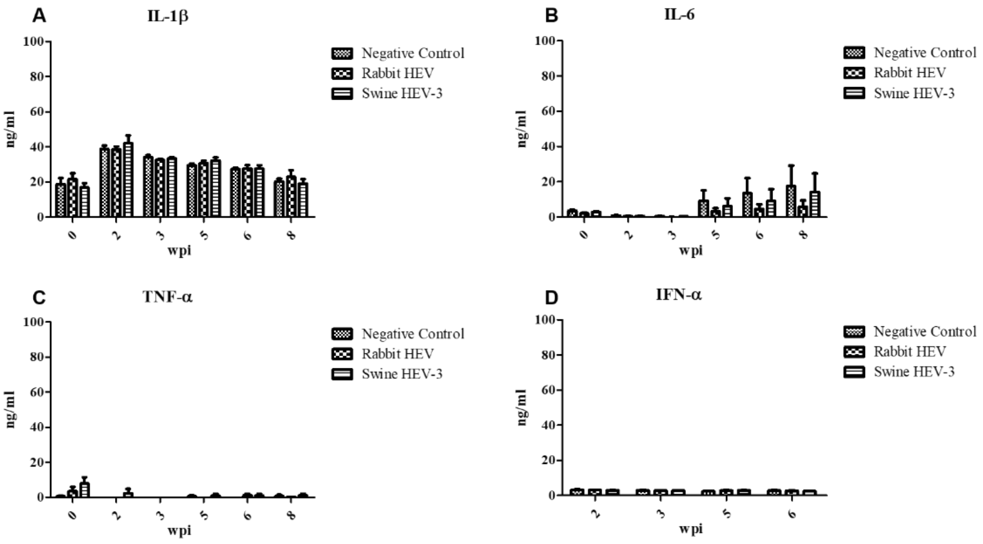

Supplementary Figure. 1. Levels of proinflammatory cytokines were measured after viral challenges with serum samples.
